# Public Health in a Global Context

**DOI:** 10.3389/fpubh.2013.00009

**Published:** 2013-04-16

**Authors:** Joav Merrick

**Affiliations:** ^1^National Institute of Child Health and Human DevelopmentJerusalem, Israel; ^2^Health Services, Division for Intellectual and Developmental Disabilities, Ministry of Social Affairs and Social ServicesJerusalem, Israel; ^3^Division of Pediatrics, Hadassah Hebrew University Medical CenterJerusalem, Israel; ^4^Kentucky Children's Hospital, University of KentuckyLexington, KY, USA

## Introduction

Public health and public health issues are not new to us. Hippocrates (460–377 BCE), a Greek physician born in 460 BC on the island of Cos (Kos) in Greece has became known as the founder of medicine and he was regarded as the greatest physician of our time. He based his medical practice on observations and on the study of the human body. He held the belief that illness had a physical and a rational explanation. He rejected the views of his time that considered illness to be caused by superstitions and by possession of evil spirits and disfavor of the gods and we can even trace the idea of public health back to him with his statement (1):

*Whoever would study medicine aright must learn of the following subjects. First he must consider the effect of each of the seasons of the year and the differences between them. Secondly he must study the warm and the cold winds, both those which are common to every country and those particular to a particular locality. Lastly, the effect of water on the health most not be forgotten*.

Plague or the Black Death has killed many people over the last several thousands of years, but understanding and preventing disease on a larger scale is something that really first came into focus during the beginning of the 1800, when public health as a modern concept emerged. Several personalities come to mind during this period of our history and they are all relevant to the emerging of public health as a specific entity within medicine in our time.

Edward Anthony Jenner (1749–1823) was an English family physician and scientist from Berkeley, Gloucestershire, who was the pioneer of smallpox vaccine, a disease that killed about 20% of infected persons. On May 14, 1796, Jenner inoculated James Phipps, an 8-year-old boy who was the son of Jenner's gardener. He scraped pus from cowpox blisters on the hands of Sarah Nelmes, a milkmaid who had caught cowpox from a cow called Blossom and inoculated Phipps in both arms that day, subsequently producing a fever and some uneasiness, but no full-blown infection. Later, he injected Phipps with variolous material, the routine method of immunization at that time, but no disease followed. The boy was later challenged with variolous material and again showed no sign of infection. Jenner can therefore be called “the father of immunology” and his work was really something that had tremendous public health effects resulting in vaccinations of whole populations and eventually eradication of smallpox.

Peter Ludvig Panum (1820–1885) was a Danish physiologist and pathologist, who in 1846 was chosen by the government to undertake research of a measles epidemic on the Faroe Islands. As a result of his investigations he published a classic public health report titled “Observations made during the epidemic of measles on the Faroe Islands in the year 1846.” Later he studied with Rudolf Virchow at the University of Würzburg (1851), and with Claude Bernard in Paris (1852–1853). From 1855 to 1864 he was professor at the University of Kiel, but afterward relocated to the University of Copenhagen as professor of physiology, where he spent the remainder of his career and today the Panum Institute forms the core of the University of Copenhagen Medical School (2).

Panum spend time with Rudolph Carl Virchow (1821–1902), who was a German doctor, anthropologist, pathologist, prehistorian, biologist, and later politician. He is considered one of the founders of social medicine and public health and his famous statement is still valid today:

*Medicine is a social science, and politics is nothing else but medicine on a large scale. Medicine, as a social science, as the science of human beings, has the obligation to point out problems and to attempt their theoretical solution: the politician, the practical anthropologist, must find the means for their actual solution… The physicians are the natural attorneys of the poor, and social problems fall to a large extent within their jurisdiction*.

With the cholera pandemic that devastated Europe between 1829 and 1851 emerged the science of epidemiology founded by the English physician John Snow (1813–1858). Snow believed in the germ theory of disease as opposed to the prevailing miasma theory. The germ theory of disease had not yet been developed, so Snow did not understand the mechanism by which the disease was transmitted. His observation of the evidence led him to discount the theory of foul air. He first publicized his theory in an essay “On the mode of communication of cholera” in 1849, followed by a more detailed report in 1855 incorporating the results of his investigation of the role of the water supply in the Soho epidemic of 1854 (3). By talking to local residents he identified the source of the outbreak as the public water pump on Broad Street (now Broadwick Street) and he persuaded the local council to disable the well pump by removing its handle, which ended the outbreak.

But public health really came into existence by end 1800 with the discoveries of Louis Pasteur (1822–1895), a French chemist and microbiologist, who can be called one of the founders of medical microbiology. He is remembered for his breakthroughs in the causes and preventions of diseases and his work reduced mortality from puerperal fever and he created the first vaccines for rabies and anthrax. His experiments supported the germ theory of disease. He was best known to the general public for inventing a method to treat milk and wine in order to prevent it from causing sickness, a process that came to be called pasteurization.

## What is Public Health?

Charles-Edward Amory Winslow (1877–1957), an American bacteriologist, who in 1915 founded the Yale Department of Public Health within the Yale Medical School defined public health as:

*The science and art of preventing disease, prolonging life, and promoting health through the organized efforts and informed choices of society, organizations, public and private, communities and individuals*.

The Institute of Medicine (IOM) in a 1988 publication (4) defined the mission of public health as

*The fulfillment of society's interest in assuring the conditions in which people can be healthy*.

and together with various public health associations in the United States formulated 10 essential elements that must be in place to create and sustain a healthy community (3):

- Monitor health status to identify community health problems
- Diagnose and investigate health problems and health hazards in the community
- Inform, educate, and empower people about health issues
- Mobilize community partnership to identify and solve health problems
- Develop policies and plans that support individual and community health efforts
- Enforce laws and regulations that protect health and ensure safety
- Link people to needed personal health services and assure the provision of health care when otherwise unavailable
- Assure a competent public health and personal health care workforce
- Evaluate effectiveness, accessibility, and quality of personal and population-based health services
- Research for new insights and innovative solutions to health problems.

## Public Health Achievements in the Twentieth Century

In the twentieth century one of the effects of improvements in public health has been an increased life expectancy in the developed world, but we have also seen other achievements. The CDC (Centers for Disease Control and Prevention) in Atlanta looked at landmarks in research, service, and intervention in public health in the developed world and published short reports on each topic of the 10 greatest achievements from 1900 to 1999 in the United States (5):

- Vaccination
- Motor-vehicle safety
- Safer workplaces
- Control of infectious diseases
- Decline in deaths from coronary heart disease and stroke
- Safer and healthier foods
- Healthier mothers and babies
- Family planning
- Fluoridation of drinking water.

## Looking at 2013

We can really be proud of our achievements, but let us look at where we are working today and again CDC has helped us focus on issues relevant today and also with a global view. The 13 public health issues of importance in 2013 are (6):

- Healthcare-associated infections
- Human immunodeficiency virus (HIV)
- CDC vital signs
- Public health grand rounds
- Million hearts^™^
- TIPS from former smokers
- Newborn screening
- Food safety
- Heads-up program
- Children's mental health
- Clinical preventive services for children and adolescents
- Preventing parasitic diseases
- Global efforts to prevent violence against children.

## Looking Globally

We live in a global village these days, where health disparities exist between and within nations and reducing these disparities are the interest of public health globally.

At a 2009 meeting at the CDC (Centers for Disease Control and Prevention) in Atlanta (7, 8) the vision for public health surveillance in the twenty-first century was discussed and global public health surveillance was found critical for the identification and prevention of emerging and reemerging diseases, both for infectious and non-communicable diseases. In the United States and elsewhere public health surveillance has evolved from monitoring infectious diseases to tracking the occurrence of many non-infectious conditions, such as injuries, birth defects, chronic conditions, disability, mental illness, illicit drug use, environmental and occupational exposures to health risks. The meeting identified six major concerns that must be addressed by the public health community to advance public health surveillance in the twenty-first century:

- Lexicon, definitions, and conceptual framework for public health surveillance
- Global health surveillance
- Roles of information sciences and technological advances in public health surveillance
- Public health surveillance work force of the future
- Accessing and using data for public health surveillance: legal, policy, ethical, regulatory, and practical concerns related to data sharing
- Analytical challenges for emerging public health surveillance.

## A Few Missing Items

Maybe because of the region I live in and maybe also because of the work that I am doing every day I find two things missing in the discussions on global public health. One topic that comes to my mind is terrorism in general, war and bioterrorism in particular.

The other concern is chronic disease and disability, which need more focus from the public health community in order to facilitate integration, better service provision, and eventually living with a disability with a good quality of life in spite of the disability.

## Looking Ahead

We need to apply what we have learned and know now in order to reduce health inequality in our nations in the future, we need to use advanced technology to improve health and address new health issues as they emerge over time, intervene, and minimize adverse effects.

Public health is community health and health care is vital to all of us some of the time, but public health is vital to all of us all of the time.
